# Splenic Artery Pseudoaneurysm Management With Cyanoacrylate Glue: A Case Report

**DOI:** 10.7759/cureus.65926

**Published:** 2024-08-01

**Authors:** Sudhanshu Tonpe, Himandri Warbhe, Pankaj Banode, Shubham Waghulkar, Shubham Dugad, Manasa Suryadevara, Swaroop Reddy Guggella

**Affiliations:** 1 Department of Interventional Radiology, Jawaharlal Nehru Medical College, Datta Meghe Institute of Higher Education and Research, Wardha, IND; 2 Department of Respiratory Medicine, Jawaharlal Nehru Medical College, Datta Meghe Institute of Higher Education and Research, Wardha, IND; 3 Radiodiagnosis, Jawaharlal Nehru Medical College, Datta Meghe Institute of Higher Education and Research, Wardha, IND; 4 Radiology, Sainath Hospital and Prime Diagnostics, Ananthapur, IND

**Keywords:** melena, hematochezia, abdominal pain, cyanoacrylate glue, pseudoaneurysm, splenic artery

## Abstract

We present the case of a 40-year-old female who presented with abdominal pain, hematochezia, and melena for the past week and was diagnosed with a pseudoaneurysm emanating from the mid-splenic artery. The patient was managed with endovascular cyanoacrylate glue embolization, resulting in the complete resolution of an impending catastrophic hemorrhagic shock.

## Introduction

Splenic artery pseudoaneurysms after aortoiliac aneurysms are the second most common abdominal aneurysms, constituting around 60% of all aneurysms [1,2]. Overall, 7% of individuals diagnosed with splenomegaly or portal hypertension exhibit a splenic artery aneurysm, while around 10% of those with a splenic artery aneurysm manifest symptoms of portal hypertension [3]. Conventional management comprises surgical simple ligation or resection of the aneurysm. Nevertheless, in recent years, endovascular techniques have been performed in growing numbers. Prior research focused on addressing endovascular therapies for splenic artery aneurysms has examined thrombin, coil embolization, gel foam detachable balloons, or a combination of these approaches [4,5]. Surgical resection of splenic artery aneurysm results in morbidity of roughly 1%-2% and mortality of 10% [6]. Here, we present the case of a patient managed with endovascular instillation of cyanoacrylate glue within the pseudoaneurysm sac.

## Case presentation

**Figure 1:**
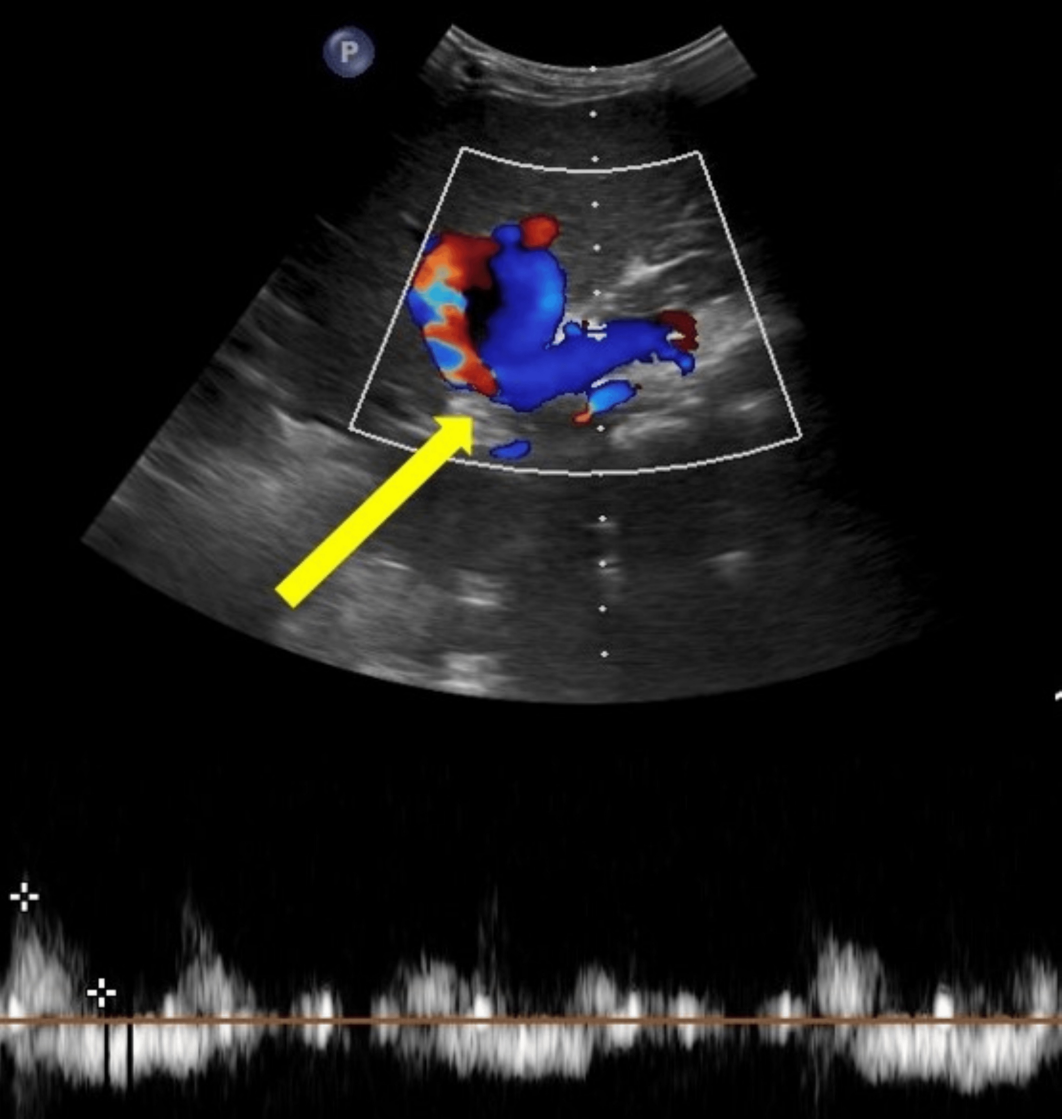
Ultrasound of the spleen demonstrating the yin yang sign (yellow arrow) on color Doppler in the splenic artery, suggestive of a pseudoaneurysm.

A 40-year-old female was admitted to the hospital with chief complaints of hematochezia, abdominal pain, and melena for the past week. On examination, the patient had splenomegaly and tenderness in the epigastric region. The patient was vitally stable. She had reduced blood hemoglobin levels of 5.5 g/dL on lab investigation. The patient underwent a diagnostic ultrasound, which demonstrated splenomegaly and a pseudoaneurysm in the splenic artery (Figure 1).

**Figure 2:**
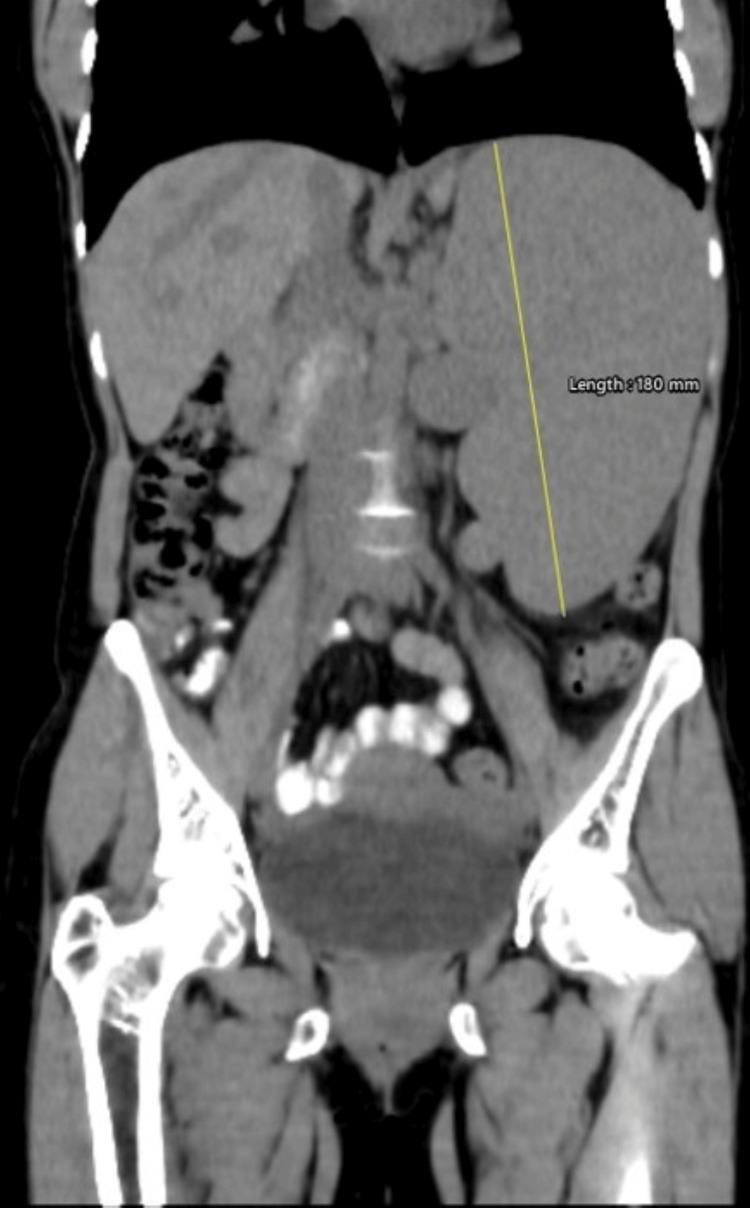
Coronal reformat of the non-contrast abdomen demonstrating splenomegaly.

**Figure 3:**
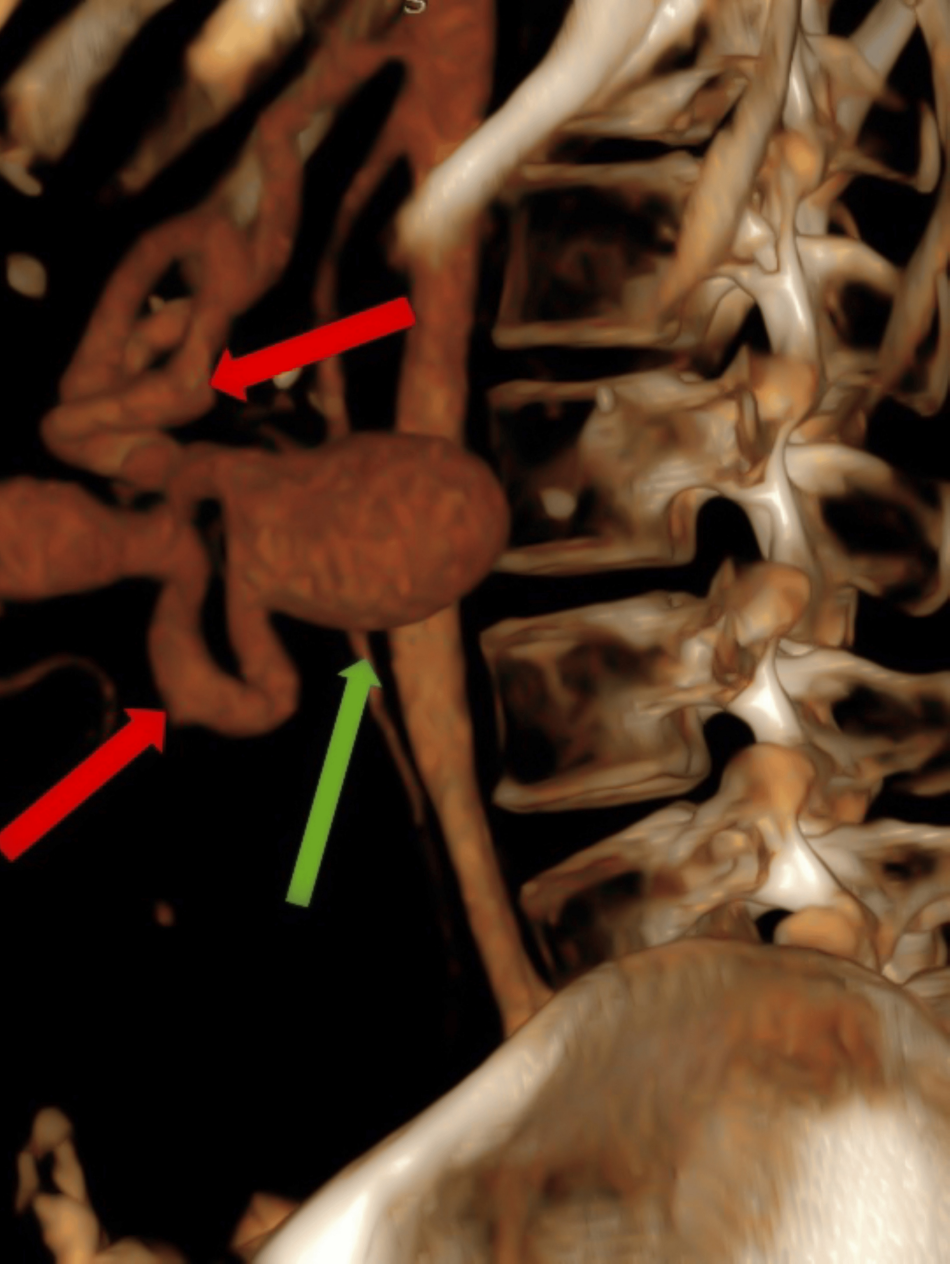
Three-dimensional reformat of the abdomen at the level of the spleen shows an aneurysmal outpouching (green arrow) in the mid-splenic artery (red arrows).

**Figure 4:**
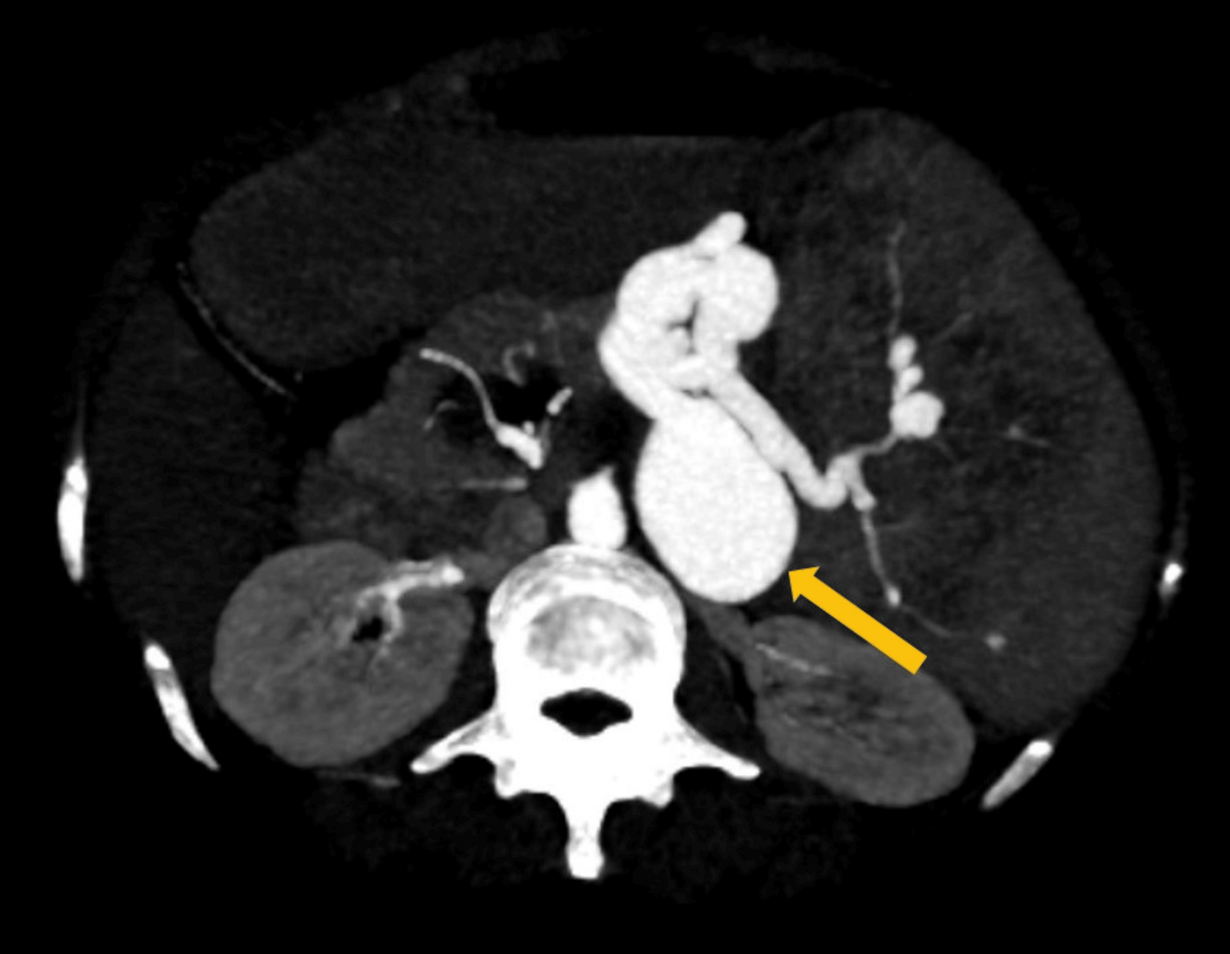
Contrast-enhanced CT in the maximum intensity projection demonstrating an outpouching in the mid-splenic artery (orange arrow).

An upper gastrointestinal endoscopy was performed on the subsequent day, and the patient had no significant ulcerations or bleeding from the upper gastrointestinal tract. The patient underwent a contrast-enhanced CT, which confirmed splenomegaly and pseudoaneurysm ultrasound findings along the splenic artery. The spleen measured 180 mm in the craniocaudal extent (Figure 2). The splenic artery pseudoaneurysm measured 43 × 32 mm (Figures 3, 4).

After a multidisciplinary meeting with the referring physician, radiologist, and interventional radiologist, the decision was made to pursue endovascular management using cyanoacrylate after vaccination with the *Haemophilus influenza* type b vaccine, pneumococcal vaccine, and meningococcal vaccine.

**Figure 5:**
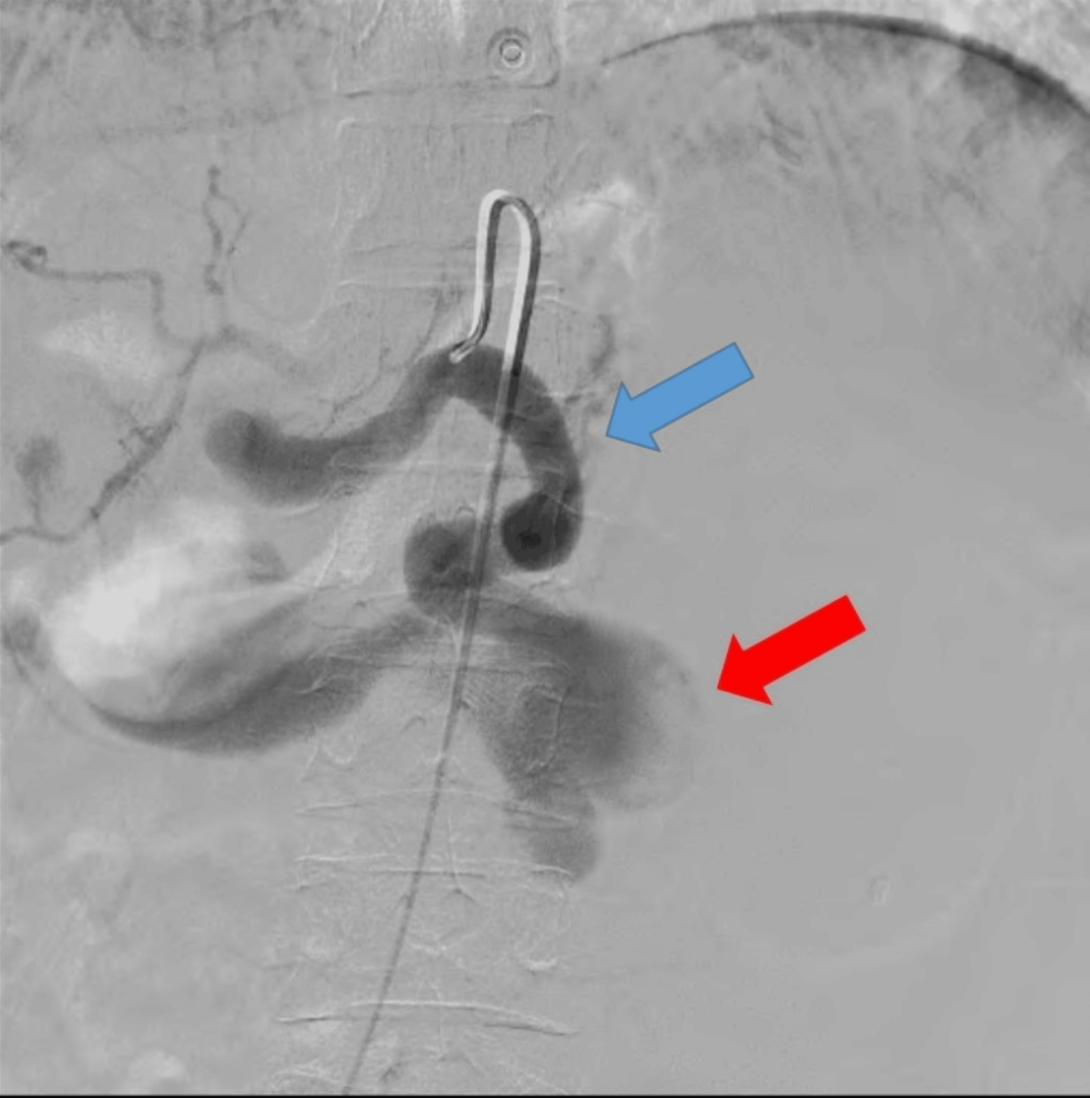
Angiography of the celiac trunk demonstrating an aneurysmal outpouching (red arrow) emanating from the mid portion of the splenic artery (blue arrow).

An angiography via the right femoral route using a 5 Fr cobra catheter was performed. The celiac artery was selectively cannulated, demonstrating an aneurysmal outpouching emanating from the middle portion of the splenic artery (Figure 5).

**Figure 6:**
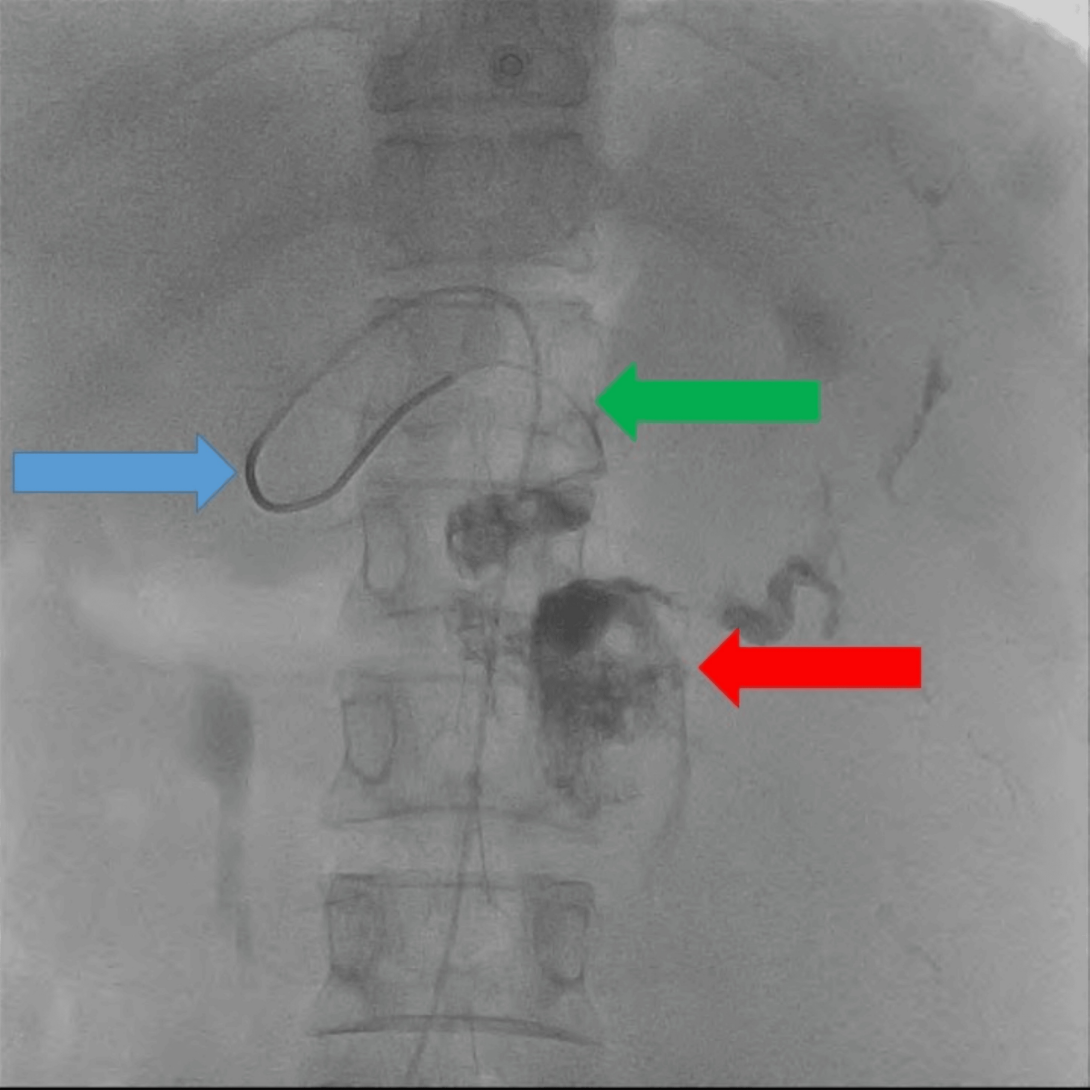
Fluoroscopic image demonstrating glue cast within the aneurysmal sac (red arrow). The microcatheter (green arrow) was introduced co-axially from the diagnostic catheter (blue arrow) into the aneurysm.

A 2.3 Fr microcatheter was coaxially introduced in the aneurysmal sac, and the pseudoaneurysm was embolized with 3 mL of 33% N-butyl cyanoacrylate glue mixed in lipiodol (Figure 6).

**Figure 7:**
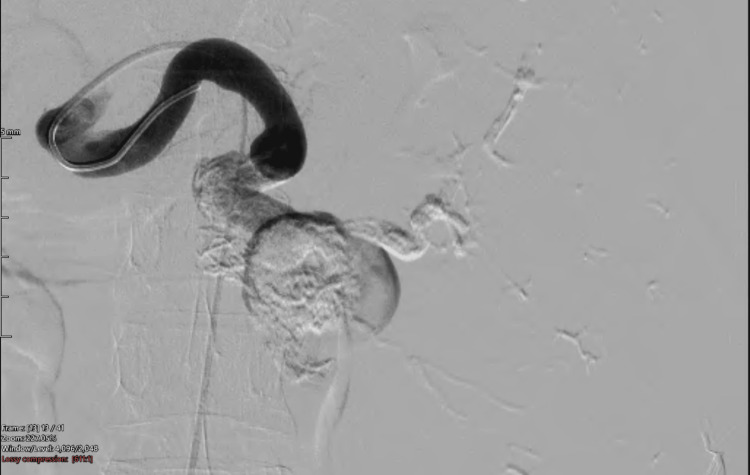
Angiography of the splenic artery demonstrating complete exclusion of the aneurysm with glue cast in the aneurysmal sac.

This concentration of glue and volume was based on the operator’s prior experience. The glue injection was performed using a 5 mL lewer lock syringe and controlled hand injection. Before the introduction of the glue within the microcatheter, the microcatheter system was meticulously prepared by flushing the catheter with roughly 5 mL of 5% dextrose, a non-ionic solution, to arrest impetuous contact of the cyanoacrylate glue concoction with ionic saline and blood and thus prevent untimely polymerization of glue enclosed in the microcatheter. After the aneurysm was completely filled with glue, the microcatheter was removed with a jerk from the diagnostic catheter. This was done to prevent the microcatheter from adhering to the glue cast inside the pseudoaneurysm. The cobra catheter was flushed with 10 mL of normal saline solution at least five times to prevent residual glue and thrombi from entering the bloodstream and causing non-target embolization. The post-embolization angiography demonstrated complete exclusion of the aneurysm (Figure 7).

**Figure 8:**
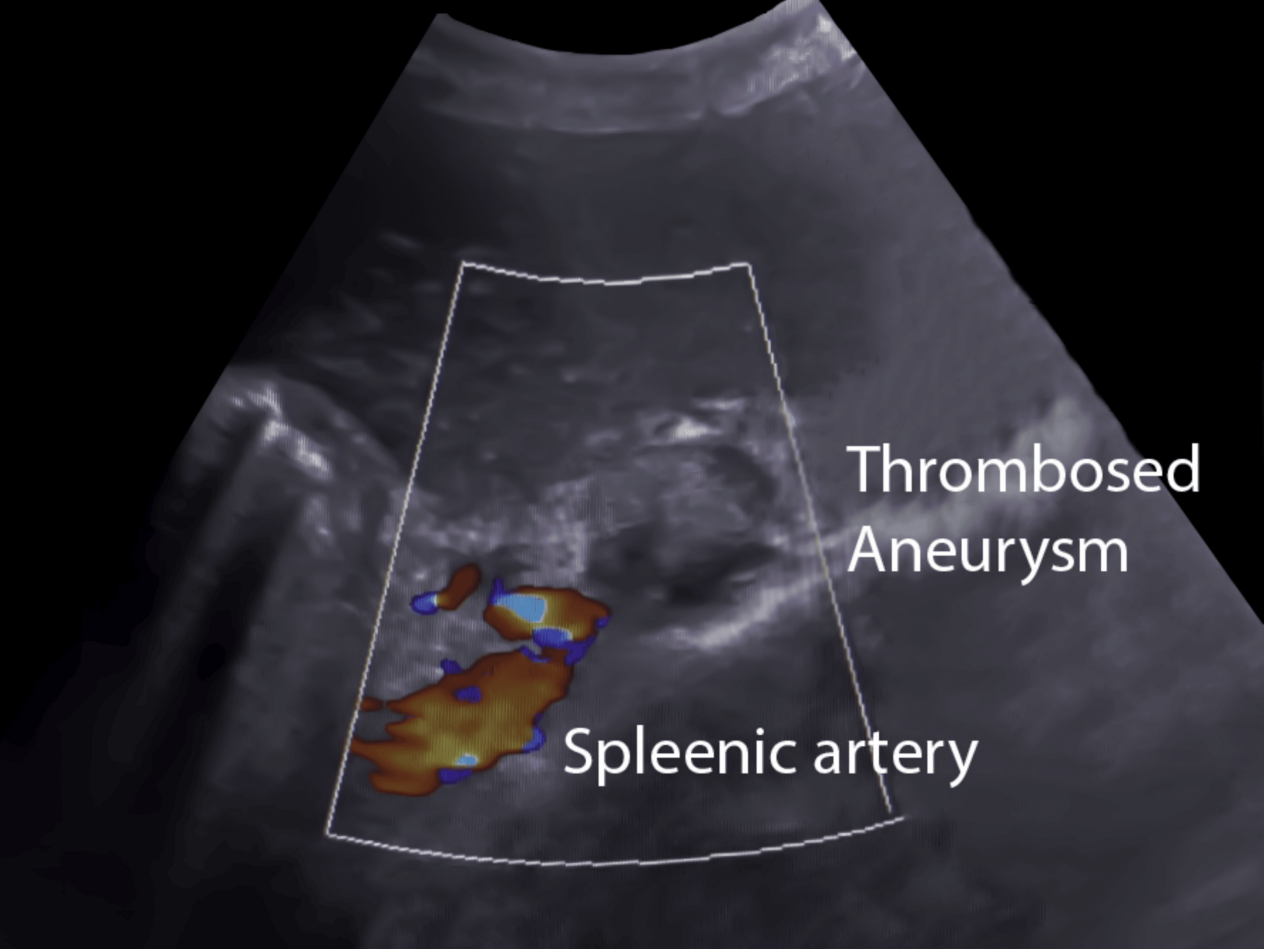
Ultrasound with color Doppler on the second day of the procedure showing complete thrombosis of the aneurysm.

In the intensive critical care unit, the patient was observed for 24 hours. The patient experienced abdominal pain and fever, which was managed conservatively with intravenous non-steroidal anti-inflammatory drugs. The patient was asymptomatic and discharged on the second day. Follow-up sonography on the second day of the procedure showed complete obliteration of the aneurysm, and there were no signs of splenic infarctions (Figure 8).

**Figure 9:**
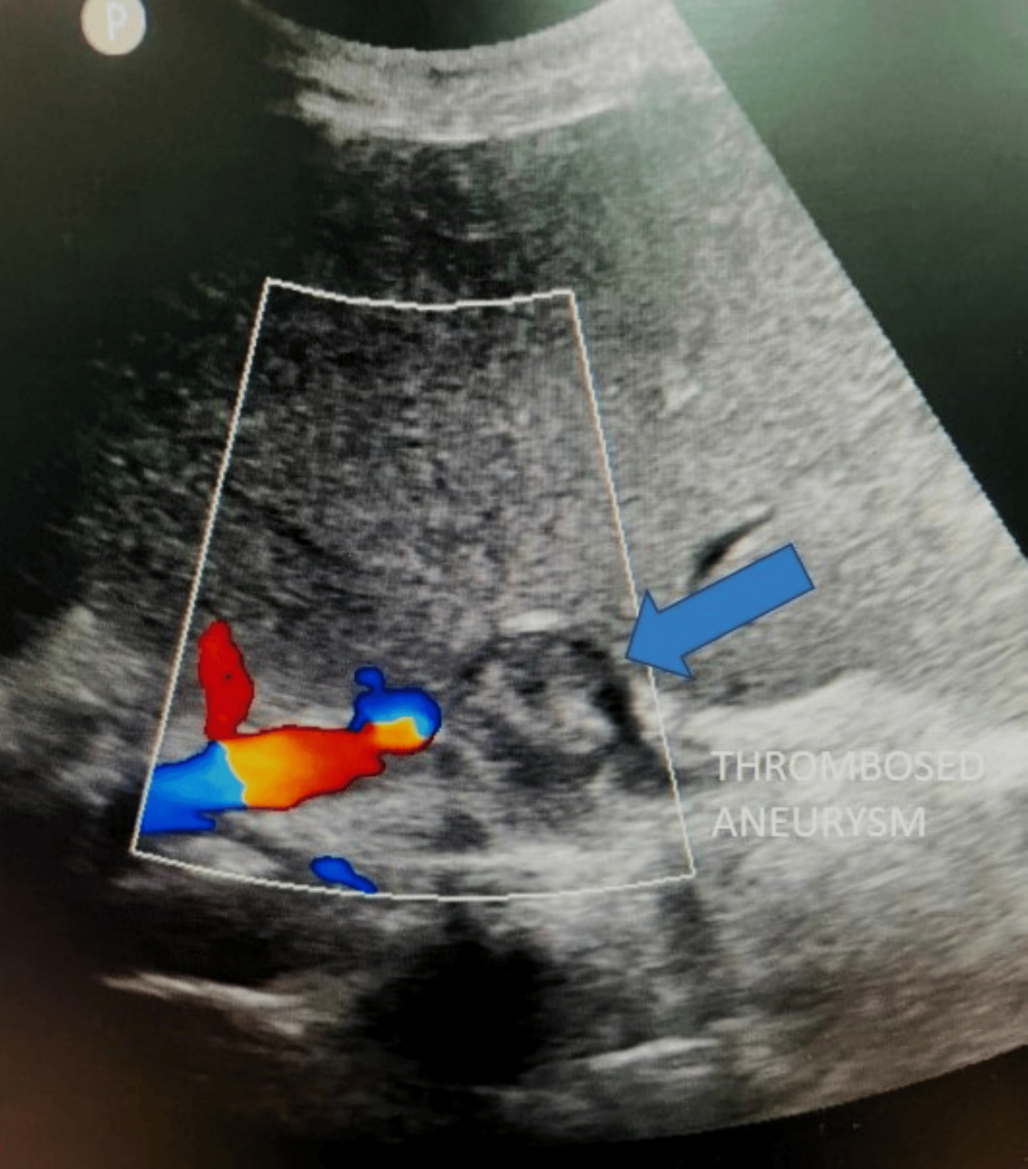
Ultrasound with color Doppler on the sixth-month follow-up showing complete thrombosis of the aneurysm with a reduction in the size of the aneurysm (blue arrow).

The abdominal pain, hematochezia, and melena resolved within a day, and the patient was on follow-up. On her six-month follow-up, the patient’s hemoglobin level was 9 g/dL. The color Doppler of the aneurysm showed no vascularity. The spleen was heterogeneous, suggesting partial infarction and mild reduction in size compared to the preoperative ultrasound (Figure 9).

## Discussion

Dotter and Goldman first described endovascular glue embolization with isobutyl 2-cyanoacrylate in treating upper gastrointestinal bleeding, pelvic hemorrhage, and selective renal artery occlusion [7,8]. Conventional management comprises surgical simple ligation or resection of the aneurysm. Endovascular management of splenic artery pseudoaneurysm with the use of stainless steel coils was first reported by Uflacker and Diehl [9]. Other procedures that have gained acceptance include embolization with micro-coils, laparoscopic surgery, and stent-graft technique [4,5,10,11]. In this case report, we discuss using cyanoacrylate glue to embolize splenic pseudoaneurysms. As the patient was unfit for surgery and the potential benefit of treating the entire diseased splenic artery and the expertise in handling cyanoacrylate glue by the interventional radiologist, the multidisciplinary team decided to proceed with endovascular management of this aneurysm. Coils and plugs were not used in our case as the objective was the exclusion of the pseudoaneurysm and not splenic infarction.

Cyanoacrylate glue rapidly polymerizes on contact with ionic solutions such as blood and saline, requiring the operator to have specialized training. The polymerization rate is adjusted with a change in the concentration of the ratio of cyanoacrylate and lipodol. The potential complication of an erroneous mixture may result in non-target proximal vascular or distal embolization. Non-target embolization can also occur with wrong injection rates. The other potential complications include abscess, rupture, septicemia, and pneumonia. This can be reduced with effective pain control, strict aseptic precautions, and careful post-embolization follow-up. Cyanoacrylate glue embolization can also treat hemobilia, visceral arteriovenous malformations, and neuro arteriovenous malformations [12]. Although most interventional radiologists are well-versed in using detachable balloons, coils, and particles such as polyvinyl alcohol and gelatin sponges, few have experience with liquid adhesives such as cyanoacrylate glue.

## Conclusions

Cyanoacrylate glue embolization by the endovascular technique should be the management methodology of choice in the treatment of splenic artery aneurysms and is a viable alternative to surgery and detachable balloons, coils, and particles such as polyvinyl alcohol and gelatin sponges due to its potential benefit of treating the entire diseased splenic artery without resulting in splenic infarctions. All interventional radiologists should be well versed in the potential applications of and handling cyanoacrylate glue. The follow-up imaging and clinical improvement demonstrate the benefits of glue embolization in the case of an unruptured splenic artery aneurysm.

## References

[1] Stanley JC, Thompson NW, Fry WJ (1970). Splanchnic artery aneurysms. Arch Surg.

[2] Dave SP, Reis ED, Hossain A, Taub PJ, Kerstein MD, Hollier LH (2000). Splenic artery aneurysm in the 1990s. Ann Vasc Surg.

[3] Shanley CJ, Shah NL, Messina LM (1996). Common splanchnic artery aneurysms: splenic, hepatic, and celiac. Ann Vasc Surg.

[4] DeRoover A, Sudan D (2001). Treatment of multiple aneurysms of the splenic artery after liver transplantation by percutaneous embolization and laparoscopic splenectomy. Transplantation.

[5] McDermott VG, Shlansky-Goldberg R, Cope C (1994). Endovascular management of splenic artery aneurysms and pseudoaneurysms. Cardiovasc Intervent Radiol.

[6] Trastek VF, Pairolero PC, Bernatz PE (1985). Splenic artery aneurysms. World J Surg.

[7] Radek A, Maciejczak A, Zajgner J (1996). [Embolization of AVM's of thoracic spinal cord with histoacryl glue]. Neurol Neurochir Pol.

[8] Siddhartha W, Parmar H, Shrivastav M, Limaye U (2000). Endovascular glue embolisation of intercostal arteriovenous fistula: a non-surgical treatment option. J Postgrad Med.

[9] Uflacker R, Diehl JC (1982). Successful embolization of a bleeding splenic artery pseudoaneurysm secondary to necrotizing pancreatitis. Gastrointest Radiol.

[10] Matsumoto K, Ohgami M, Shirasugi N, Nohga K, Kitajima M (1997). A first case report of the successful laparoscopic repair of a splenic artery aneurysm. Surgery.

[11] Yoon HK, Lindh M, Uher P, Lindblad B, Ivancev K (2001). Stent-graft repair of a splenic artery aneurysm. Cardiovasc Intervent Radiol.

[12] Kim BS, Do HM, Razavi M (2004). N-butyl cyanoacrylate glue embolization of splenic artery aneurysms. J Vasc Interv Radiol.

